# Association Between Body Mass Index and Hypertension Among Indigenous Adults in Canada: A Cross-Sectional Analysis of the 2022 Canadian Community Health Survey

**DOI:** 10.7759/cureus.111994

**Published:** 2026-07-03

**Authors:** Amna Waqar, Akinyele Oladimeji, Joseph E Igetei, Redvers Kadzungwa, Adaeze E Uzozie, Uwanmwende Omenai, Sarah Waseem, Onize Ekome, Emmanuella E Ogelebor

**Affiliations:** 1 Obstetrics and Gynaecology, Ayub Medical College, Abbottabad, PAK; 2 Family Medicine, Alberta Health Services, Edmonton, CAN; 3 General Medicine, International University of Health Sciences, Basseterre, KNA; 4 Epidemiology and Public Health, Boston University School of Public Health, Boston, USA; 5 General Medicine, University of Nigeria Teaching Hospital, Enugu, NGA; 6 Psychiatry, Federal Neuropsychiatric Hospital, Benin, NGA; 7 General Medicine, Dow University of Health Sciences, Karachi, PAK; 8 Rehabilitation Science, McMaster University, Hamilton, CAN; 9 Mental Health, Braxia Health, Mississauga, CAN; 10 Internal Medicine, Kwame Nkrumah University of Science and Technology, Kumasi, GHA

**Keywords:** body mass index, canada, cardiovascular risk, hypertension, indigenous health, obesity

## Abstract

Background

Hypertension and obesity remain important public health concerns among Indigenous populations in Canada because of their association with cardiovascular disease and related chronic conditions. Limited recent national evidence has examined the relationship between body mass index and hypertension among Indigenous adults in Canada.

Objective

To examine the association between body mass index classification and hypertension among Indigenous adults in Canada using data from the 2022 Canadian Community Health Survey.

Methods

This cross-sectional study analyzed data from the 2022 Canadian Community Health Survey Public Use Microdata File. The study included 1,632 Indigenous adults aged 18 years and older, representing a weighted population of 607,892 individuals. Survey-weighted descriptive statistics and multivariable logistic regression analyses were performed while accounting for the complex survey design and bootstrap variance estimation. Hypertension was the outcome variable, while body mass index classification was the primary exposure variable.

Results

Among participants with hypertension, 92,384 (87.03%) were classified as overweight or obese, compared with 351,946 (70.14%) among those without hypertension. Adults aged 50-64 years had significantly higher odds of hypertension compared with adults aged 18-34 years (adjusted odds ratio (aOR)=9.54, 95% confidence interval (CI): 2.70-33.69, p<0.001). Participants aged 65 years or older also had higher odds of hypertension (aOR=13.59, 95% CI: 3.68-50.14, p<0.001). High cholesterol was significantly associated with hypertension (aOR=2.40, 95% CI: 1.23-4.70, p=0.011). Overweight or obese participants had higher odds of hypertension compared with underweight or normal weight participants; however, the association did not reach statistical significance after adjustment for demographic, behavioral, and clinical factors (aOR=2.15, 95% CI: 0.95-4.88, p=0.066).

Conclusion

This study highlights the burden of hypertension among Indigenous adults in Canada. Older age and high cholesterol were significantly associated with hypertension. Although overweight or obese participants demonstrated higher odds of hypertension, the association did not reach statistical significance after adjustment. Further longitudinal studies are needed to better understand the relationship between body mass index and hypertension among Indigenous populations in Canada.

## Introduction

Worldwide, hypertension is a major public health challenge and one of the leading risk factors for cardiovascular disease, stroke, chronic kidney disease, and premature mortality [1]. Obesity, typically assessed via body mass index (BMI), is one of the most common modifiable risk factors related to higher blood pressure and the development of hypertension [2,3]. Excessive fat contributes to poor vascular function, insulin resistance, inflammatory states, and increased workload on the heart, which increase the risk of developing hypertension [4]. In Canada, hypertension remains a significant public health issue that affects millions of adults and contributes significantly to the chronic disease burden and health-related quality of life [5]. The increasing incidence of overweight and obesity among Canadian adults was reported in national surveillance reports over the last several decades [6].

In Canada, Indigenous communities (First Nations, Métis, and Inuit) experience a disproportionate rate of obesity, hypertension, diabetes and cardiovascular disease compared to non-Indigenous Canadians [7,8]. The relationship between the higher rates of disease in these communities and the historical and structural inequities that result from colonization, food insecurity, poverty, poor housing, limited access to health care, and geographic isolation is well established [9,10]. Continuing to emphasize the social determinants of health and their role in chronic disease burden is essential because these factors contribute to poorer health outcomes among Indigenous populations [11]. Beyond hypertension, social determinants of health are increasingly recognized as important contributors to adverse cardiovascular outcomes, including heart failure, atrial fibrillation, and cardiovascular mortality, with growing evidence demonstrating that social adversity is associated with poorer clinical outcomes across a range of cardiovascular conditions [11,12]. Previous research has also demonstrated that Indigenous populations experience a high burden of obesity-related comorbidities, which are associated with an increased risk of developing cardiovascular disease [13,14].

Many studies conducted in Canada have determined that an increase in adiposity is associated with increased blood pressure in Indigenous adults [15]. Low levels of physical activity, sedentary lifestyles, tobacco use, and unhealthy food habits could also lead to higher incidences of obesity and the risk of developing hypertension among indigenous persons [16,17]. Furthermore, the lack of access to preventative health care services and delays in the diagnosis of chronic diseases disproportionately affect Indigenous individuals [18]. In addition, emerging data suggest that the COVID-19 pandemic may have further exacerbated health inequities and worsened health outcomes for chronic diseases in Canada's vulnerable populations [19].

Although previous studies have documented high rates of obesity, hypertension, and cardiovascular disease among Indigenous populations in Canada [7,13,14], much of the available evidence is derived from regional studies, specific Indigenous communities, or older datasets [13-15]. Consequently, contemporary nationally representative evidence examining the relationship between body mass index classification and hypertension among Indigenous adults remains limited [20,21]. The 2022 Canadian Community Health Survey provides an opportunity to evaluate this relationship using recent national data and to better characterize the current patterns of cardiovascular risk among Indigenous populations in Canada [5,22]. Therefore, the objective of this study was to examine the association between body mass index classification and hypertension among Indigenous adults in Canada using data from the 2022 Canadian Community Health Survey.

## Materials and methods

Study design and data source

This study used a cross-sectional analytical design based on data from the 2022 Canadian Community Health Survey, a nationally representative health survey conducted by Statistics Canada [22]. The survey collects information related to health status, chronic conditions, health behaviors, and healthcare utilization among individuals aged 12 years and older living in Canada. The Canadian Community Health Survey applies a complex multistage sampling design incorporating stratification, clustering, and unequal probability sampling methods to generate representative population estimates. Public Use Microdata File data from the 2022 survey cycle were used for the present analysis. The study focused on Indigenous adults residing in Canada to examine the association between body mass index and hypertension.

Study population

**Figure 1:**
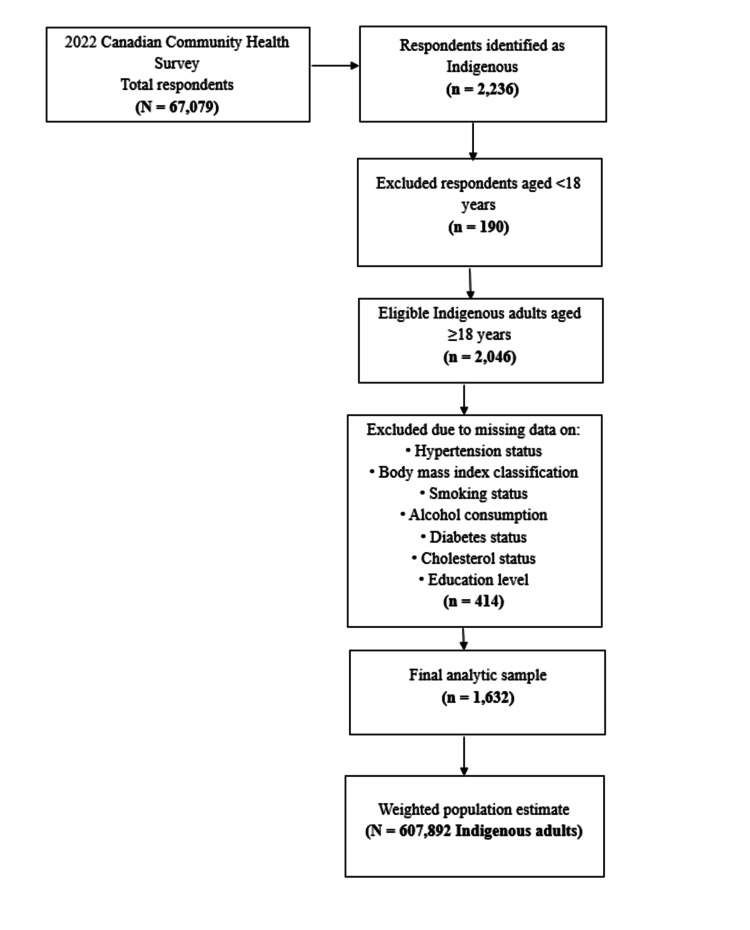
Participant selection flowchart for Indigenous adults included in the analysis using the 2022 Canadian Community Health Survey

The 2022 Canadian Community Health Survey included 67,079 respondents sampled from across Canada. For the present study, respondents identified as Indigenous were selected from the full dataset, resulting in an initial subgroup of 2,236 individuals. The analysis was subsequently restricted to adults aged 18 years and older because the adjusted adult body mass index classification variable was only applicable to adult respondents. After applying the age restriction, the eligible study population included 2,046 Indigenous adults. Participants with missing data on hypertension status, body mass index classification, smoking status, alcohol consumption, diabetes status, cholesterol status, or education level were excluded using complete case analysis. The final analytic sample consisted of 1,632 unweighted respondents, representing a weighted population estimate of 607,892 Indigenous adults in Canada as shown in Figure 1.

Variables and measures

The primary outcome variable was hypertension status, derived from the survey item assessing whether respondents had been diagnosed with high blood pressure by a health professional. Hypertension was coded as a binary variable categorized as yes or no. The primary exposure variable was body mass index classification based on the adjusted adult body mass index variable available in the Canadian Community Health Survey dataset. Body mass index was categorized into two groups: underweight or normal weight, and overweight or obese, including obesity classes I to III groups to ensure sufficient observations within each category and improve the stability of survey-weighted regression estimates in the Indigenous study population. Sociodemographic covariates included age group, sex, and education level. Age was categorized into 18 to 34 years, 35 to 49 years, 50 to 64 years, and 65 years or older. Education level was categorized as less than secondary education, secondary education, and post-secondary education. Behavioral covariates included smoking status and alcohol consumption. Smoking status was categorized as current smoker, former smoker, and never smoker. Alcohol consumption was categorized as regular drinker, occasional drinker, and non-drinker. Clinical covariates included diabetes status and high blood cholesterol status, both coded as binary variables. Covariates included in the multivariable logistic regression model were selected a priori based on an established evidence of their associations with both body mass index and hypertension, as well as their potential role as the confounding factors and their availability within the Canadian Community Health Survey dataset. Demographic, behavioral, and clinical variables were therefore included to better estimate the independent association between body mass index classification and hypertension.

Missing data

Several study variables contained missing data. Body mass index classification had the highest proportion of missingness (7.87%), followed by smoking status (5.87%), education level (5.08%), diabetes status (3.08%), alcohol consumption (0.49%), hypertension status (0.49%), and high cholesterol status (0.34%). Variables including age group, sex, Indigenous identity, and survey weight had no missing data. Complete-case analysis was considered appropriate because missingness for individual variables was generally low and there was no evidence to suggest systematic patterns of missing data within the analytic sample. Nevertheless, the possibility of selection bias resulting from the exclusion of participants with incomplete data cannot be entirely excluded.

Statistical analysis

All analyses accounted for the complex survey design of the Canadian Community Health Survey using sampling weights, strata, clusters, and bootstrap replicate weights as recommended by Statistics Canada. Weighted descriptive statistics were used to summarize participant characteristics according to hypertension status. Descriptive estimates were presented as weighted frequencies and column percentages. Group comparisons by the outcome variable were not performed because the use of bootstrap replicate weights does not support reliable inference from standard statistical tests in descriptive cross-tabulations. Multivariable survey weighted logistic regression analysis was conducted to evaluate the association between body mass index classification and hypertension after adjustment for age group, sex, education level, smoking status, alcohol consumption, diabetes, and high cholesterol. Adjusted odds ratios and 95% confidence intervals were reported. Multicollinearity among independent variables was assessed using variance inflation factors. Variance inflation factor values ranged from 1.02 to 1.93, with a mean variance inflation factor of 1.40, indicating no evidence of substantial multicollinearity. Statistical significance was defined using a two-sided p-value less than 0.05. All analyses were conducted using Stata version 18 (Statacorp, College Station, TX, USA) [23].

Ethical considerations

This study used publicly available de-identified data from the 2022 Canadian Community Health Survey Public Use Microdata File provided by Statistics Canada. The dataset contains no directly identifiable personal information. Ethical approval for the original survey data collection was obtained by Statistics Canada. Secondary analysis of publicly available anonymized survey data is generally considered exempt from additional institutional ethics review according to prevailing research ethics guidelines.

## Results

Table 1 presents the sociodemographic, behavioral, and clinical characteristics of Indigenous adults according to hypertension status in the 2022 Canadian Community Health Survey.

**Table 1:** Sociodemographic and Clinical Characteristics of Indigenous Adults by Hypertension Status, Canadian Community Health Survey (CCHS) 2022 (N=607,892 ,n=1632) Values are presented as weighted counts and column percentages derived from the Canadian Community Health Survey 2022. Estimates account for the complex survey design using sampling weights and bootstrap variance estimation. Formal group comparisons were not performed because the use of bootstrap replicate weights does not support reliable inference from standard statistical tests in descriptive cross tabulations. The - (dash) appearing in columns means intentionally left blank. All the estimates were generated using Stata version 18 [23].

Variable	No Hypertension (N=501,749)	Hypertension (N=106,143)
Sex, n (%)	–	–
Male	251,730 (50.17%)	57,974 (54.62%)
Female	250,019 (49.83%)	48,169 (45.38%)
Age group, n (%)	–	–
18-34 years	246,843 (49.20%)	8,869 (8.36%)
35-49 years	136,946 (27.29%)	23,362 (22.01%)
50-64 years	81,914 (16.33%)	45,516 (42.88%)
≥65 years	36,046 (7.18%)	28,396 (26.75%)
Education level, n (%)	–	–
Less than secondary	32,596 (6.50%)	15,674 (14.77%)
Secondary	107,391 (21.40%)	38,736 (36.49%)
Post-secondary	361,762 (72.10%)	51,733 (48.74%)
Body Mass Index classification, n (%)	–	–
Underweight/Normal weight	149,803 (29.86%)	13,759 (12.97%)
Overweight/Obese	351,946 (70.14%)	92,384 (87.03%)
Smoking status, n (%)	–	–
Current smoker	117,609 (23.44%)	33,984 (32.02%)
Former smoker	107,208 (21.37%)	34,631 (32.63%)
Never smoker	276,932 (55.19%)	37,528 (35.35%)
Alcohol consumption, n (%)	–	–
Regular	301,527 (60.10%)	63,268 (59.61%)
Occasional	105,853 (21.10%)	15,115 (14.24%)
Non-drinker	94,369 (18.80%)	27,760 (26.15%)
Diabetes, n (%)	–	–
No diabetes	480,761 (95.82%)	82,199 (77.44%)
Diabetes	20,988 (4.18%)	23,944 (22.56%)
High cholesterol, n (%)	–	–
No high cholesterol	456,929 (91.07%)	68,932 (64.94%)
High cholesterol	44,820 (8.93%)	37,211 (35.06%)

The findings show that participants with hypertension were generally older than those without hypertension, with 45,516 (42.88%) aged 50-64 years and 28,396 (26.75%) aged 65 years or older, compared with 81,914 (16.33%) and 36,046 (7.18%), respectively, among those without hypertension. Overweight or obesity was more common among participants with hypertension (92,384 (87.03%) vs. 351,946 (70.14%)). Diabetes (23,944 (22.56%) vs. 20,988 (4.18%)) and high cholesterol (37,211 (35.06%) vs. 44,820 (8.93%)) were also more prevalent among participants with hypertension. Overall, these descriptive findings indicate a greater burden of cardiometabolic risk factors among Indigenous adults with hypertension. Detailed weighted frequencies and percentages are presented in Table 1.

Table 2 presents the multivariable logistic regression analysis examining factors associated with hypertension among Indigenous adults in Canada.

**Table 2:** Multivariable Logistic Regression Analysis of Factors Associated with Hypertension Among Indigenous Adults in Canada, Canadian Community Health Survey (CCHS) 2022 Adjusted odds ratios (OR) with 95% confidence intervals (CI) were estimated using survey-weighted logistic regression models accounting for the complex sampling design of the Canadian Community Health Survey 2022, including bootstrap variance estimation. Asterisk (*) denotes statistical significance at p<0.05. Adjusted for sex, age group, education level, smoking status, alcohol consumption, diabetes, and high cholesterol. All the estimates were generated using Stata version 18 [23].

Variable	Adjusted OR	95% CI	p-value
Body Mass Index classification			
Overweight/Obese (Class I–III) vs Underweight/Normal weight	2.15	0.95-4.88	0.066
Sex			
Female vs male	0.83	0.45-1.53	0.553
Age group			
35-49 years vs 18-34 years	4.1	1.13-14.83	0.031*
50-64 years vs 18-34 years	9.54	2.70-33.69	<0.001*
≥65 years vs 18-34 years	13.59	3.68-50.14	<0.001*
Education level			
Secondary education vs Less than secondary	0.97	0.29-3.19	0.955
Post-secondary education vs Less than secondary	0.43	0.14-1.34	0.146
Smoking status			
Former smoker vs current smoker	0.74	0.30-1.80	0.507
Never smoker vs current smoker	0.62	0.27-1.47	0.282
Alcohol consumption			
Occasional drinker vs regular drinker	0.69	0.33-1.44	0.32
Non-drinker vs regular drinker	0.66	0.30-1.45	0.299
Diabetes			
Diabetes vs No diabetes	2.15	0.89-5.19	0.089
High cholesterol			
High cholesterol vs No high cholesterol	2.4	1.23-4.70	0.011*

The adjusted analysis showed that age was strongly associated with hypertension. Compared with participants aged 18-34 years, adults aged 35-49 years had higher odds of hypertension (adjusted OR 4.10, 95% CI 1.13 to 14.83, p=0.031). Adults aged 50-64 years had adjusted odds of hypertension that were approximately nine times higher than those aged 18-34 years (adjusted OR 9.54, 95% CI 2.70 to 33.69, p<0.001). Participants aged 65 years or older also had markedly higher odds of hypertension (adjusted OR 13.59, 95% CI 3.68 to 50.14, p<0.001).

Participants classified as overweight or obese had higher odds of hypertension compared with those classified as underweight or normal weight (adjusted OR 2.15, 95% CI 0.95-4.88), although this association was not statistically significant (p=0.066). Female sex was not significantly associated with hypertension compared with male sex (adjusted OR 0.83, 95% CI 0.45-1.53, p=0.553). Education level, smoking status, and alcohol consumption were also not significantly associated with hypertension after adjustment.

Participants with diabetes had higher odds of hypertension compared with those without diabetes (adjusted OR 2.15, 95% CI 0.89-5.19), although the association was not statistically significant (p=0.089). High cholesterol was significantly associated with hypertension. Participants with high cholesterol had more than twice the odds of hypertension compared with those without high cholesterol (adjusted OR 2.40, 95% CI 1.23 to 4.70, p=0.011).

## Discussion

This study examined the association between body mass index classification and hypertension among Indigenous adults in Canada using nationally representative data from the 2022 Canadian Community Health Survey. A higher proportion of participants with hypertension were classified as overweight or obese compared with those without hypertension. In the adjusted analysis, overweight or obese participants had more than twice the odds of hypertension; however, the association did not reach statistical significance after adjustment for demographic and clinical factors. Older age and high cholesterol were independently associated with hypertension, whereas diabetes demonstrated elevated odds but was not statistically significant after adjustment.

The attenuation of the association between body mass index classification and hypertension after adjustment may reflect the influence of demographic and clinical factors that are strongly associated with both obesity and hypertension. Adjustment for age, diabetes, and high cholesterol may have attenuated the observed association between body mass index classification and hypertension. Because diabetes and high cholesterol may lie on the causal pathway linking excess body weight and hypertension, some degree of overadjustment cannot be excluded and should be considered when interpreting these findings. In addition, dichotomization of body mass index into underweight/normal weight and overweight/obese categories may have reduced statistical power and limited the ability to detect dose-response relationships across the full spectrum of body mass index classifications. Although obesity is a well-established risk factor for hypertension, the findings of the present study should therefore be interpreted cautiously. Furthermore, several adjusted estimates were accompanied by relatively wide confidence intervals, suggesting limited precision for some subgroup estimates. These findings should therefore be interpreted cautiously and confirmed in larger studies.

The strong association between increasing age and hypertension is consistent with previous studies showing that vascular aging, arterial stiffness, and cumulative cardiometabolic risk contribute to elevated blood pressure among older adults [1-4]. Similarly, the independent association between high cholesterol and hypertension is consistent with evidence demonstrating the clustering of cardiometabolic risk factors among individuals at increased cardiovascular risk [4]. Although diabetes was not independently associated with hypertension after adjustment, the elevated odds observed are consistent with previous Canadian studies reporting frequent coexistence of diabetes and hypertension among Indigenous populations [12,18].

Previous Canadian studies have documented a high burden of obesity, hypertension, diabetes, and cardiovascular disease among Indigenous populations [7,13,17]. While obesity and hypertension commonly coexist, the lack of a statistically significant independent association after adjustment in the present study suggests that the relationship may be influenced by other demographic and cardiometabolic factors. These findings highlight the importance of considering multiple cardiovascular risk factors rather than body mass index alone when assessing hypertension risk among Indigenous adults.

Social and structural factors remain important contributors to cardiovascular health among Indigenous populations. Geographic isolation, long distances to healthcare facilities, limited availability of healthcare providers, reduced access to healthy foods, and socioeconomic disadvantage may reduce opportunities for hypertension prevention, early diagnosis, and long-term management [7,8,11]. Continued efforts to improve culturally appropriate chronic disease prevention, equitable access to healthcare services, and community-based cardiovascular risk reduction remain important for improving cardiovascular health outcomes among Indigenous communities.

Strengths and limitations

This study has several strengths. The analysis used nationally representative survey data and appropriately accounted for the complex survey design through the application of sampling weights and bootstrap variance estimation. In addition, the study specifically examined Indigenous adults, a population that remains underrepresented in national cardiovascular research.

Several limitations should also be considered. Hypertension, diabetes, high cholesterol, smoking, alcohol consumption, height, and weight were based on self-reported survey responses and may therefore be subject to recall, reporting, and misclassification bias. Body mass index was derived from self-reported height and weight, which may have resulted in some misclassification of weight status. The dichotomization of body mass index into two categories may have reduced statistical power and limited the ability to detect dose-response relationships across standard body mass index classifications. Although complete-case analysis was performed because missingness for individual variables was relatively low, exclusion of participants with incomplete data may have introduced selection bias. Several adjusted estimates were accompanied by relatively wide confidence intervals, suggesting limited precision for some subgroup comparisons and should therefore be interpreted with caution. Important variables, including dietary sodium intake, antihypertensive medication adherence, household income, healthcare access, and objectively measured physical activity, were unavailable or incomplete and may have resulted in residual confounding. Finally, the cross-sectional design precludes an assessment of temporal relationships or causal inference. Future longitudinal studies using objectively measured anthropometric and clinical data are needed to better understand the relationship between body mass index and hypertension among Indigenous populations in Canada.

## Conclusions

This study provides contemporary nationally representative evidence describing the relationship between body mass index classification and hypertension among Indigenous adults in Canada. Older age and high cholesterol were independently associated with hypertension, whereas the association between overweight or obesity and hypertension did not reach statistical significance after adjustment. These findings contribute to the limited national evidence on cardiovascular risk among Indigenous populations and support the need for further longitudinal studies to better understand hypertension risk in this population. Future longitudinal studies are needed to further examine the relationship between body mass index and hypertension and to investigate additional social, behavioral, and healthcare-related factors that may influence cardiovascular risk.
